# The regulatory effects of water probiotic supplementation on the blood physiology, reproductive performance, and its related genes in Red Tilapia (*Oreochromis niloticus X O. mossambicus*)

**DOI:** 10.1186/s12917-024-04190-w

**Published:** 2024-08-07

**Authors:** El-Sayed Hemdan Eissa, Abdel-Fattah M. El-Sayed, Basma M. Hendam, Sara F. Ghanem, Heba E. Abd Elnabi, Yasmin M. Abd El-Aziz, Sameh A. Abdelnour, Moaheda E.H. Eissa, Hagar Sedeek Dighiesh

**Affiliations:** 1 Fish Research Centre, Faculty of Agricultural Environmental Sciences, Arish University, El-Arish, 45511 Egypt; 2 Oceanography Department, Faculty of Science, Alexandria University, Alexandria, Egypt; 3 Department of Husbandry and Development of Animal Wealth, Mansoura University, Mansoura, Egypt; 4 National Institute of Oceanography and Fisheries (NIOF), Cairo, Egypt; 5 Department of Fish Resources and Aquaculture, Faculty of Environmental Agricultural Sciences, Arish University, El-Arish, Egypt; 6 Zoology Department, Faculty of Science, Port-Said University, Port Fouad, 42526 Egypt; 7 Department of Animal Production, Faculty of Agriculture, Zagazig University, Zagazig, 44511 Egypt; 8 Biotechnology Department, Fish Farming and Technology Institute, Suez Canal University, Ismailia, 41522 Egypt; 9 Department of Aquaculture, Faculty of Fish Resources, Suez University, Suez, 43512 Egypt

**Keywords:** Probiotic, Water quality, Red Tilapia, Blood health, Sex hormones, Histology and Gene expression

## Abstract

Probiotics are becoming increasingly popular as eco-friendly alternatives in aquaculture. However, there is limited research on their impacts on the reproductive efficiency of Red Tilapia (*Oreochromis niloticus* x *O. mossambicus*) broodstock. Therefore, this experiment aimed to explore the combined effects of selective probiotics *Bacillus subtilis* and *B. licheniformis* (BSL; 1:1) added to water on blood hematology, serum metabolites, gonadal histology, reproductive performance, and reproductive associated genes in Red Tilapia broodstock. Tilapia broodfish weighing 140–160 g were stocked in four treatment groups: control (T0), and the other three groups were added different levels of BSL to the water as follows: T1 (0.01 g/m^3^), T2 (0.02 g/m^3^), and T3 (0.03 g/m^3^), respectively. Results indicate that BSL administration significantly improved RBCs, hemoglobin, hematocrit, MCH, and MCHC, with the highest improvement seen in the T3 group (*P* < 0.05). BSL added to the fish water significantly enhanced serum protein fractions (total protein, albumin, and globulins), while AST, ALT, ALP, creatinine, uric acid, and glucose were significantly diminished in a dose-dependent way (*P* < 0.05). Adding 0.02–0.03 g/ m^3^ of BSL resulted in higher antioxidant status (superoxide dismutase and catalase) compared to other groups (*P* < 0.05). Testosterone levels were higher in T3 than in other groups (*P* < 0.05). All female hormones (LH, FSH, estradiol, and progesterone) were substantially augmented by the addition of BSL. Additionally, the BSL groups exhibited higher GSI, HSI, VSI (male only), egg diameter (mm), mean number of fry/fish, and mean fry weight (g) compared to the control group (*P* < 0.05). Expression of reproductive-associated genes (*vasa*,* nanos1a*,* nanos2*,* dnd1*,* pum1*,* AMH*, and *vtg*) were significantly up-regulated in the gonads of fish in the 0.03 g/m^3^ treatment. The histological gonadal structure exhibited that BSL improved gonad maturation in both genders of Tilapia fish. Overall, adding a mixture of *B. subtilis* and *B. licheniformis* (0.03 g/m^3^ water) can accelerate reproductive performance in Red Tilapia through up-regulation of reproductive genes and enhance the health profile.

## Introduction

Aquaculture’s sustainability depends on the effective utilization of aquafeeds and the implementation of robust aquaculture health management practices. Aquaculture contributes to approximately 50% of the world’s total fish production, solidifying its position as the fastest-growing sector within the industry [1, 2]. Moreover, it plays a significant part in providing sustainable income opportunities and contributing to global food security [3]. In Egypt, there are numerous fish species that inhabit its water resources. The country’s diverse aquatic ecosystems support a wide variety of fish, including Nile tilapia, catfish, and mullet [4]. These fish play a crucial role in the local economy and provide a vital source of protein for the population. The aquaculture industry in Egypt is an important sector that contributes to the country’s food security and economic development. However, local production rarely meets domestic demand, leading the country to rely on imports to cover the shortage [2]. Tilapias are a globally farmed group of fish, with a production of approximately 6.7 million tons in 2023. This industry is valued at over 14.1 billion US dollars [5]. As omnivorous fish, tilapias can host both beneficial and harmful bacteria in their gastrointestinal tract, culture water, and sediment. Some examples of bacteria found in the gastrointestinal tract of Nile Tilapia fish include *Lactobacillus farciminis*,* Lactobacillus coryniformis*,* Lactobacillus brevis*,* Lactobacillus collinoides*,* Bacillus* sp., and others. *Bacillus* sp., P. *Fluorescens*, L. *brevis*, and *L. collinoides* are commonly abundant in the fish’s gut [3, 6].

Probiotics have emerged as a promising alternative strategy for preventing infectious diseases [7, 8]. In aquaculture, probiotics offer numerous benefits, such as improving water quality, enhancing digestion, and boosting fish growth and immune response [9–11]. Probiotics can enhance feed efficiency in aquatic animals by increasing the activity of digestive enzymes and maintaining a healthy balance of intestinal microbes. This leads to better nutrient absorption and utilization, as well as improved reproductive system function [12, 13]. Probiotic supplementation also increases appetite and the digestibility of organisms [9]. *B. subtilis* and *B. licheniformis* bacteria are important probiotic additives for maintaining the normal growth and functions of aquatic animals’ by providing vitamins, nutrients, and producing digestive enzymes. These factors positively effects on feed utilization, nutritional absorption, and growth performance [14]. *Bacillus spp.* have various positive ways, such as promoting better nutrient utilization, production and secretion of exogenous enzymes, and enhancing gut microbiota to support intestinal physiological functions [10, 15, 16]. Therefore, fishes fed with different Bacillus species have shown improve growth indices [17]. Additionally, altering the harmful intestinal microbiota composition to favour a greater proportion of beneficial bacterial communities can support adaptive and innate functions and promote intestinal integrity in the host [3].

Probiotic mechanisms include actions to inhibit pathogen growth, production of various ingredients (e.g., organic acids, bacteriocins, and volatile compounds), competition for adherence sites and nutrients [18, 19], and enhancement of innate immune responses (e.g., respiratory burst activities, lysozyme enhancement) and interactions with natural killer cells, leukocytes, and phagocytes [11, 19]. An appropriate and balanced diet not only provides the principal and necessary components for better fish growth but also commonly includes feed supplements such as herbal extracts, probiotics, and symbiotics to boost the immune system and growth rate [17]. Probiotic addition has been shown to enhance antioxidant capacity, digestive enzymes, and immune function in Nile tilapia fish [6, 8, 20]. Additionally, both serum and mucosal surfaces’ immunoglobulin M (IgM) levels have been found to play an essential role in defending against numerous pathogenic organisms that infect cultured fish [21, 22]. One important attribute of Bacillus species is their ability to form spores, which allows them to withstand the heat generated during feed palletization [16, 17, 23]. These spores also enable the bacteria to survive the adverse environment of the fish’s stomach and colonize the intestines, where they can multiply and produce various beneficial digestive enzymes such as amylase, lipase, and protease [23–25]. Additionally, probiotics’ molecular mechanism of action involves influencing the expression and regulation of different genes [15, 26, 27]. Therefore, the authors of the current study discovered that increasing levels of *B. subtilis* and *B. licheniformis* have many substantially beneficial consequences on the physiology, blood health and reproductive performance of red tilapia. With this backdrop, the experiment was conducted to determine the effect of graded levels of water probiotics, *B. subtilis* and *B. licheniformis* on hematological variables, reproductive ability, and expression of reproductive-related genes in Red Tilapia broodstock (*O. niloticus* x *O. mossambicus*).

## Materials and methods

### Fish and experimental design

This experiment was performed at the Fish Research Centre, Arish University, North Sinai, Egypt. Adult male and female hybrid red tilapia (*O. niloticus x O. mossambicus*) weighing 140–160 g were housed in concrete tanks with a volume of 3 × 4 × 0.8 m³. A total of 480 fish were used in this experiment. The fish were divided into four groups, with each treatment consisting of 120 fish (three tanks, 40 fish in each tank). The fish were stocked in triplicates at a ratio of 1 male to 3 females per cubic meter, with a total density of forty fish per tank (around 860 g/m^3^). The fish were acclimated to the trial culture conditions for fifteen days. Air stones were provided in the tanks throughout the trial, and a light cycle of 12 h light and 12 h dark was maintained. Four treatments were included: a control group (T0), and three groups (T1, T2 and T3) with varying levels of *B. subtilis* and *B. licheniformis* added to the water. The treatment fish groups (2.5% of the total biomass) were labeled as follows: T0 (0 g/m^3^), T1 (0.01 g/m^3^), T2 (0.02 g/m^3^), and T3 (0.03 g/m^3^). The fish were fed an extruded diet from ALLER AQUA FEED (https://www.aller-aqua.com/) with 30% crude protein, 5.2% crude fat, 5.8% total ash, and 4.8% crude fiber. Each morning, before the first feeding, the fish feces and waste were siphoned, and approximately 10% of the pond water was replaced with dechlorinated water of similar temperature. Every day, the doses of *B. subtilis* and *B. licheniformis* were adjusted according to the rate of water changes. At the end of the experiment, the fish from each tank were collected, tallied, weighed, and the weights and survival rate were documented. The fishpond was then cleaned, and the fish were prepared for the spawning period. Thirty ripe females and ten ripe males were placed in the culture tanks for 20 days. During this period, the reproduction capability and spawning performance were measured.

### Water quality parameters

The water elements such as salinity (g/L), temperature (°C), pH, dissolved oxygen (DO, mg/L), total ammonia nitrogen (TAN, mg/L), and ammonia (mg/L) were monitored twice a week employing the YSI-556 multi-parameter method (YSI Inc., Yellow Springs, OH, USA) to assess water quality.

### Blood sampling

Before the final harvest, all fish were fasted for approximately 12 h. Subsequently,5 fish per tank were anesthetized using amino-benzoic acid (120 mg/L, Sigma-Aldrich, Germany) for blood sample collection. The blood samples were obtained for hemato-biochemical and other physiological parameters. Blood samples were captured from the caudal vein using sterilized needles and separated into subsamples. The first part was stored in heparinized tubes for the hematology parameters analysis. While, the second part was stored in non-heparinized tubes and left to coagulate at room temperature, following the method described by [25] for serum separation. The blood was then centrifuged at 4000 rpm for 10 min to separate the serum, which was subsequently stored at -20 °C for further analysis.

### Blood hematology assessment

Red blood cells (RBCs) were counted using the method described by [28] with a Bright-Line Hemocytometer (Neubauer enhanced, Germany). Hemoglobin (Hb) levels were measured calorimetrically, as outlined by [29]. Hematocrit (Hct) was calculated following the method of [30]. The levels of MCV (mean corpuscular volume), MCHC (mean corpuscular hemoglobin concentration), and MCH (mean corpuscular hemoglobin) were determined according to [28].

### Serum metabolites assays

The serum total protein fraction (total protein and albumin) was determined using kits provided by Diamond Diagnostics Company. Globulin concentration was calculated using the difference method between total protein and albumin. Kidney related biomarkers such as uric acid, creatinine, and urea were assess according to the method of [31] using kits provided by Biocompare company (South San Francisco, United States). Glucose levels were determined by the colorimetric glucose oxidase technique of [32]. The activities of ALT (alanine aminotransferase), AST (aspartate aminotransferase), and ALP (alkaline phosphatase) were measured using an automated analyzer (Abbott Alcyon 300, USA) in accordance with the Pars Azmon Kit’s protocol (Pars Azmon, Iran). The “hydroxylamine method” was used to determine superoxide dismutase (SOD) activity [33], while the “visible light method” used for catalase (CAT) activity [34]. Steroid female hormones such as estradiol (E2, MBS700179), progesterone (P, MBS2602842), luteinizing (LH, MBS283097), and follicle-stimulating (FSH, MBS281137) hormones were determined using commercial ELISA kits as explained by [35]. Testosterone (T, MBS933475) hormone was assessed using quantitative competitive method by ELISA kit. All kits used for steroids hormones were provided by the MyBiosource company (San Diego, USA).

### Organosomatic indices

The total body length (T.L) in centimeters and weight (W) in grams were recorded for 30 fish in each group (15 males and 15 females). The liver, gut, and gonads of 30 fish (5 males and 5 females/ tank) per group were removed and weighed. The hepatosomatic index (HSI), viscerasomatic index (VSI), and gonadosomatic index (GSI) were calculated using the following equations:

$$HIS{\text{ }}\left( \% \right){\text{ }} = {\text{ }}100\left\{ \begin{gathered} liver{\text{ }}weight{\text{ }}\left( g \right)/ \hfill \\ gutted{\text{ }}body{\text{ }}weight{\text{ }}\left( g \right) \hfill \\ \end{gathered} \right\}$$

$$GSI{\text{ }}\left( \% \right){\text{ }} = {\text{ }}100{\text{ }}\left\{ \begin{gathered} gonads{\text{ }}weight{\text{ }}\left( g \right)/ \hfill \\ gutted{\text{ }}body{\text{ }}weight{\text{ }}\left( g \right) \hfill \\ \end{gathered} \right\}$$

$$VSI{\text{ }}\left( \% \right){\text{ }} = {\text{ }}100\left\{ \begin{gathered} visceral{\text{ }}weight{\text{ }}\left( g \right)/{\text{ }} \hfill \\ gutted{\text{ }}body{\text{ }}weight{\text{ }}\left( g \right) \hfill \\ \end{gathered} \right\}$$

### Egg diameter, mean number of fry/fish and mean fry weight

For 20 days, the spawning performance was monitored. Five gravid, spawn-ready females were eliminated from all tanks, gently stripped, and then subsamples of around ten eggs were randomly selected for determining the diameter of eggs (mm) [36]. Each female was returned to the appropriate tank after stripping until the end of the trial. Females in each tank were checked daily to find eggs or fry. The eggs were left in the females’ mouths until hatching and complete yolk sac absorption. The fry were then gathered from their respective females, counted, and weighed; the averages were evaluated following the method described by [37] method. By distributing the total quantity of fry in the tank by the number of female spawns, the mean number of fry per spawning was determined.

### Genes expression

#### cDNA production and total RNA extraction

Samples of testes and ovaries were collected and frozen using liquid nitrogen to analyze the expression of various reproduction-related genes. Each 50 mg of ovarian and testicular tissues was used for RNA extraction with Trizol reagent (iNtRON Biotechnology, Inc., South Korea). The RNA concentration was determined using a NanoDrop method (UV-Vis spectrophotometer, USA). The cDNA was synthesized with the Fast HiSenScript TM RH RT PreMix cDNA synthesis kit (iNtRON Biotechnology, South Korea), and the samples were kept at -20 °C for further analysis.

#### Real time qPCR (RT-PCR)

The specific primer sequences, product sizes, and GenBank accession numbers of reproduction-associated genes, namely *vasa*,* nanos1a*,* nanos2a*,* dnd1*,* pum1*,* AMH*, and *vtg* for both males and females, are listed in Table 1. The *Elf1α* gene served as a housekeeping (reference) gene for normalizing mRNA expressions. RT-PCR was performed using the SYBR Green PCR Master Mix to quantify the mRNA expression of the target genes (SensiFast™ SYBR Lo-Rox kit, Bioline). The thermocycling settings were as follows: 95 °C for 10 min, followed by 40 cycles at 94 °C for 15s, 60 °C for 1 min, and 72 °C for 20 s. The mRNA expression levels of each gene were normalized and standardized to the mRNA of *elf1α* transcripts using the 2^−ΔΔCT^ approach [38].

**Table 1:** Sequence of forward and reverse primers used for q-PCR analysis

Gene^1^	Primer sequence	Gen. bank accession no.	product size
*Vasa*	F:3’-CGATGAGATCTTGGTGGATG-5’ R:3’-CATGAGATCCCTGCCAGCAGA-5’	XM_019351277.2	175 bp
*nanos1a*	F:3’-TCTCAGGCCATACGAACACCTCG-5’ R:3’-CTCTGAGCCTGTTTGCGTCTTCG-5’	XM_003447766.4	126 bp
*nanos2*	F:3’-CGGGAAAGTTTTCTGCCCCATCC-5’ R:3’-AGAACTTGGCCCCTGTCTCCATC-5’	XM_005448855.3	140 bp
*dnd1*	F:3’-CACGGGACACGTATGAGGACATC-5’ R:3’-ATATTTGGCATACGCAAAGCCGC-5’	XM_003454288.4	119 bp
*pum1*	F: 3’-GCTAACTGGTAAGAAGTTCTGGGAA-5’ R: 3’-CGGGACACCATGATTGGCTG-5’	XM_013270654.3	135 bp
*Amh*	F: 3’-AAGCAGCGCAAACATTAACA-5’ R: 3’-GTTCCAGTCCACAACCTCCA-5’	XM_013275129.3	169 bp
*vtg*	F: 3’-CTTTCCATCCAGCCACCAAG-5’ R: 3’-CTGCAGGAGGTTGATGATGC-5’	XM_003452574.4	161 bp
*elf1α*	F: 3’-CTGGACAAACTGAAGGCTGAGCG-5’ R: 3’-AAGTCTCTGTGTCCAGGGGCATC-5’	NM_001279647.1	116 bp

^1^The target genes are *vasa*,* nanos1a*,* nanos2*,* dnd1*,* pum1*,* AMH*, and *vtg*, while elf1α is the housekeeping gene. Elongation factor 1-α (elf1α), vitellogenin (*vtg*), anti-Müllerian hormone (*AMH*), pumilio RNA binding family member 1 (*pum1*), dead end (dnd1), Nanos C2HC-Type Zinc Finger 2 (*nanos2)*, Nanos C2HC-Type Zinc Finger 1- *a* (*nanos1a)* and DEAD-box RNA helicase Vasa *(Vasa)*

### Histological analysis

The testes and ovaries of males and females were freshly removed, fixed in neutral formaldehyde (10%) for 24 h, then dehydrated with graded ethanol, and immersed in methyl benzoate for 24 h. They were then cleared in xylene, embedded in purified paraffin wax, and sectioned to a thickness of 5–7 μm using an automated microtome. The sectioned tissues were stained with hematoxylin and eosin and examined under a light microscope (Zeiss) using the method described by [39].

### Statistical analysis

Results are presented as means ± standard error (S.E.). All numerical data were checked for homogeneity of variance using Levene’s test and for normality of distribution using the Shapiro-Wilk test. The data were analyzed using SPSS software (Version 26.0; SPSS, Chicago, IL, USA) through a one-way analysis of variance (ANOVA) to determine statistical significance at a 95% confidence level. If the F values from the ANOVA test were found to be significant (*P* < 0.05), Duncan’s multiple range test was also used to compare means.

## Results

### Water quality

The administration of BSL significantly improved water quality variables (*P* < 0.05; Table 2). Total ammonia nitrogen (TAN) and NH_3_ values were reduced in a dose-dependent manner (*P* < 0.05) with the most significant decrease observed in T3. The pH values were significantly lower in the T2 and T3 treatments compared to other treatments (*P* < 0.05). Salinity levels did not vary among the groups (*P* > 0.05). T3 revealed lower dissolved oxygen (DO) levels compared to other groups (*P* < 0.05).

**Table 2:** Effect of the *Bacillus subtilis* and *B. Licheniformis* (BSL, mixture 1: 1) on water quality parameters

Parameters	Probiotics addition (g/m^3^)			
	0.0	0.01	0.03	0.03
**Temperature (°C)**	27.16 ± 0.09^b^	27.57 ± 0.12^a^	27.16 ± 0.14^b^	27.31 ± 0.20^ab^
**Salinity (g/L)**	51.33 ± 6.81	52.33 ± 3.0	52.67 ± 5.51	52.0 ± 2.64
**Dissolved oxygen (mg/L)**	7.96 ± 0.062^ab^	8.17 ± 0.12^a^	7.70 ± 0.18^b^	7.35 ± 0.20^c^
**pH**	8.16 ± 0.006^a^	8.17 ± 0.06^a^	8.13 ± 0.02^b^	8.13 ± 0.015^b^
**Total ammonia nitrogen (mg/L)**	0.86 ± 0.039^a^	0.68 ± 0.07^b^	0.53 ± 0.06^c^	0.43 ± 0.03^d^
**Ammonia (NH**_**3**,_ **mg/L)**	0.08 ± 0.03^a^	0.06 ± 0.04^b^	0.04 ± 0.02^c^	0.03 ± 0.03^c^

* The values are reported as means ± SE. Data in the same row with different superscripts indicate significant differences (*P* < 0.05), determined using ANOVA Post Hoc (Duncan test)

### Hematological and biochemical parameters

The impact of various doses of BSL (0.01, 0.02, and 0.03 g/m^3^) on hemato-biochemical parameters is shown in Table 3. The results show a significant increase in RBC counts, Hb, and Hct in T3 (*P* < 0.05) compared to other groups. MCV did not show a significant change (*P* > 0.05) with the addition of probiotics. In contrast, MCH and MCHC values were substantially increased (*P* < 0.05) in T3, with MCHC reaching its maximum value in this treatment. Besides, the highest values of albumin, total protein, and globulin (*P* < 0.05) were obtained in the *B. subtilis* and *B. licheniformis* (0.03 g/m^3^) treatment.

**Table 3:** Effect of the *B. subtilis* and *B. Licheniformis* levels (0, 0.01, 0.02, 0.03, g//m^3^) added to the water on blood hematology and serum metabolites parameters (mean ± SD) of Red Tilapia *Oreochromis spp*

Parameters^1^	Probiotics addition (g/m^3^)			
	0.0	0.01	0.02	0.03
**Hematological indices**				
RBCs ×10^6^ cell/ml	1.29 ± 0.01^b^	1.34 ± 0.03^ab^	1.39 ± 0.06^a^	1.42 ± 0.05^a^
Hb (g/dl)	6.10 ± 0.06^d^	7.18 ± 0.07^c^	7.73 ± 0.21^b^	8.02 ± 0.08^a^
MCHC (%)	22.09 ± 0.64^b^	25.31 ± 0.96^a^	26.24 ± 0.70^a^	26.47 ± 0.69^a^
MCV (fl.)	209.76 ± 2.95	211.25 ± 1.29	212.51 ± 1.71	213.10 ± 1.72
Hct (% (	26.99 ± 0.14^c^	28.38 ± 0.83^bc^	29.47 ± 1.36^ab^	30.33 ± 1.06^a^
MCH (pg)	47.44 ± 0.20^c^	53.45 ± 1.73^b^	55.63 ± 1.55^ab^	56.41 ± 1.50^a^
**Hepatic function**				
Total proteins (g/dL)	3.24 ± 0.03^d^	3.47 ± 0.02^c^	3.63 ± 0.06^b^	4.07 ± 0.06^a^
Globulin (g/dL)	1.83 ± 0.02^b^	1.84 ± 0.02^b^	2.16 ± 0.04^a^	2.20 ± 0.06^a^
Albumin (g/dL)	1.40 ± 0.04^d^	1.63 ± 0.03^b^	1.47 ± 0.04^c^	1.87 ± 0.03^a^
Glucose (mg/dL)	134.53 ± 1.12^a^	127.32 ± 1.78^b^	127.94 ± 1.56^b^	115.94 ± 1.41^c^
ALT (U/L)	47.94 ± 0.48^a^	46.98 ± 0.33^b^	46.14 ± 0.20^c^	45.18 ± 0.26^d^
AST (U/L)	125.52 ± 0.93^a^	122.52 ± 1.04^b^	118.90 ± 0.81^c^	118.09 ± 0.61^c^
ALP (U/L)	25.97 ± 0.34^a^	24.06 ± 0.16^b^	21.84 ± 0.66^c^	21.03 ± 0.58^c^
**Renal function**				
Uric acid (mg/dL)	1.75 ± 0.06^a^	1.44 ± 0.08^b^	1.13 ± 0.07^c^	0.97 ± 0.05^c^
Urea (mg/dL)	23.64 ± 0.65^a^	21.73 ± 0.56^ab^	19.66 ± 0.44^bc^	18.25 ± 1.03^c^
Creatinine (mg/dL)	61.49 ± 0.77^a^	56.12 ± 1.48^b^	50.97 ± 2.22^c^	47.95 ± 0.78^d^
**Antioxidant capacity**				
SOD (u /mg. protein)	24.97 ± 0.40^c^	29.53 ± 0.68^b^	31.90 ± 1.28^a^	33.53 ± 0.91^a^
CAT (µmol)	42.37 ± 1.40^c^	44.20 ± 0.89^b^	45.23 ± 1.44^a^	46.17 ± 1.19^a^

*The values are reported as means ± SE (*n* = 6). Data in the same row with different superscripts indicate significant differences (*p* < 0.05), determined using ANOVA Post Hoc (Duncan test). ^1^CAT, catalase; SOD, superoxide dismutase; RBCs, Red blood cells; AST, Aspartate transaminase; MCV, Mean Corpuscular Volume; Hb, hemoglobin; MCHC, mean corpuscular hemoglobin concentration; MCH, ALT; Alanine transaminase; Mean corpuscular Hemoglobin; Htc, Haematocrit. ALP, alkaline phosphatase. ^2^ Red Tilapia treated with various levels of *Bacillus subtilis* and *B. licheniformis* (0, 0.01, 0.02, 0.03, g/m^3^) added to the water

The hepatic function enzymes ALT, AST, and ALP were notably affected (*P* < 0.05) by the addition of BSL, with higher levels observed in the untreated group compared to other treatments. The probiotics-treated groups shown lower values for ALP, AST, and ALT than the control group (*P* < 0.05), indicating improved liver function. Similar trends were observed for creatinine, urea, and uric acid (*P* < 0.05), suggesting that probiotics enhanced overall fish health. In terms of antioxidant enzymes CAT and SOD, there was a significant increase (*P* < 0.05) with higher levels of B. subtilis and B. licheniformis. Both T2 and T3 groups exhibited superior values of SOD and CAT compared to other groups (*P* < 0.05).

### Reproductive hormones

Table 4 shows that the treatment with three levels of *B. licheniformis* and *B. subtilis* had a significantly higher effect (*P* < 0.05) on Red Tilapia reproductive hormones compared to the control group. Specifically, the probiotic treatment at level 3 (T3, 0.03 g/m^3^) showed significant results (*P* < 0.05) in increasing the concentrations of the hormones FSH, LH, E2, and progesterone compared to the other treatment groups. Regarding testosterone hormone parameters, the highest concentration increase was observed in the T3 treatment, while there was no significant difference among T1, T2, and the control group.

**Table 4:** Effect of the *B. subtilis* and *B. Licheniformis* levels (0, 0.01, 0.02, 0.03, g//m^3^) added to the water on male and female hormones of Red Tilapia *Oreochromis spp*

Sex Hormone^1^		Probiotics addition (g/m^3^)^2^			
		0.0	0.01	0.02	0.03
Male	T (ng/mL)	2.55 ± 0.16^b^	2.72 ± 0.18^b^	2.86 ± 0.30^b^	3.24 ± 0.08^a^
Female	LH (mIU/mL)	37.33 ± 2.52^b^	43.67 ± 4.04^b^	51.67 ± 5.13^a^	57.67 ± 1.5^a^
	FSH (mIU/mL)	53.00 ± 3.61^d^	68.67 ± 4.16^c^	77.33 ± 3.06^b^	86.00 ± 3.61^a^
	E2 (pg/mL)	104.00 ± 6.00^c^	125.67 ± 4.51^b^	147.67 ± 7.77^a^	157.33 ± 2.5^a^
	Prog (ng/mL)	1.51 ± 0.04^c^	1.73 ± 0.08^bc^	2.15 ± 0.12^b^	3.00 ± 0.53^a^

* The values are reported as means ± SE (*n* = 6). Data in the same row with different superscripts indicate significant differences (*P* < 0.05), determined using ANOVA Post Hoc (Duncan test). ^1^FSH, Follicle-stimulating hormone; Prog, progesterone; T, Testosterone; LH, luteinizing hormone; and E2, estradiol. ^2^ Red Tilapia treated with various levels of *Bacillus subtilis* and *B. licheniformis* (0, 0.01, 0.02, 0.03, g/m^3^) added to the water

### Organosomatic indexes

The findings suggest that the levels of *B. subtilis* and *B. licheniformis* positively influenced the organosomatic indexes and reproductive functions (Table 5). Both *B. subtilis* and *B. licheniformis* levels contributed to hepatic and gonadal development in both sexes compared to the control group (*P* < 0.05). The hepatosomatic index (HSI) ranged from 3.07 to 3.55% for males and 3.09–3.34% for females. The viscerosomatic index (VSI) was significantly impacted by the addition of various doses of *B. subtilis* and *B. licheniformis* in all treatments (*P* < 0.05), ranging from 9.99 to 11.06%. However, the VSI for females showed no significant effect with the addition of different levels of B. subtilis and B. licheniformis in all treatments (*P* > 0.05). The gonadosomatic index (GSI) significantly improved in all probiotic treatments (*P* < 0.05), ranging from 3.36 to 4.95% for males and 4.06–5.05% for females.

**Table 5:** Effect of the *Bacillus subtilis* and *B. licheniformis* (0, 0.01, 0.02, 0.03, g/m^3^) added to the water on reproductive performance and Organosomatic indices of Red Tilapia *Oreochromis spp*

Parameters/Sex^1^	Probiotics addition (g/m^3^)^2^			
	0.0	0.01	0.03	0.03
HSI (%)				
Male	3.07 ± 0.08^b^	3.23 ± 0.06^b^	3.55 ± 0.03^a^	3.55 ± 0.06^a^
Female	3.09 ± 0.08^b^	3.31 ± 0.08^a^	3.31 ± 0.08^a^	3.34 ± 0.04^a^
VSI (%)				
Male	9.99 ± 0.06^d^	10.31 ± 0.09^c^	10.76 ± 0.06^b^	11.06 ± 0.07^a^
Female	9.97 ± 0.33	9.55 ± 0.41	9.50 ± 0.55	9.69 ± 0.38
GSI (%)				
Male	3.36 ± 0.17^d^	3.87 ± 0.04^c^	4.45 ± 0.06^b^	4.95 ± 0.04^a^
Female	4.06 ± 0.22^d^	4.32 ± 0.07^c^	4.68 ± 0.06^b^	5.02 ± 0.12^a^
Egg diameter (mm)	1.17 ± 0.06^b^	1.51 ± 0.12^a^	1.59 ± 0.05^a^	1.69 ± 0.19^a^
Mean Number of fries/fish	1130.0 ± 19.9^d^	1271.33 ± 48.8^c^	1356.33 ± 17.6^b^	1478.33 ± 22.6^a^
Mean fry weight (g)	16.05 ± 0.13^c^	16.35 ± 0.09^b^	16.42 ± 0.08^b^	16.89 ± 0.07^a^

* The values are reported as means ± SE (*n* = 6). Data in the same row with different superscripts indicate significant differences (*P* < 0.05), determined using ANOVA Post Hoc (Duncan test). ^1^HSI, hepatosomatic index; GSI, Gonadosomatic index; and VSI, viscerosomatic index. ^2^ Red Tilapia treated with various levels of *Bacillus subtilis* and *B. licheniformis* (0, 0.01, 0.02, 0.03, g/m^3^) added to the water

### Egg diameter, mean number of fry/fish and mean fry weight

The inclusion of varying levels of *B. subtilis* and *B. licheniformis* resulted in a notable improvement in egg diameter, the average number of fry (spawning efficiency and larval production), and the average fry weight. Egg diameter varied from 1.17 mm to 1.69 mm, the mean number of fries ranged from 1130 to 1478, and the average fry weight ranged from 16.05 g to 16.89 g, as presented in Table 5.

### Reproductive development associated gene expression

**Fig. 1:**
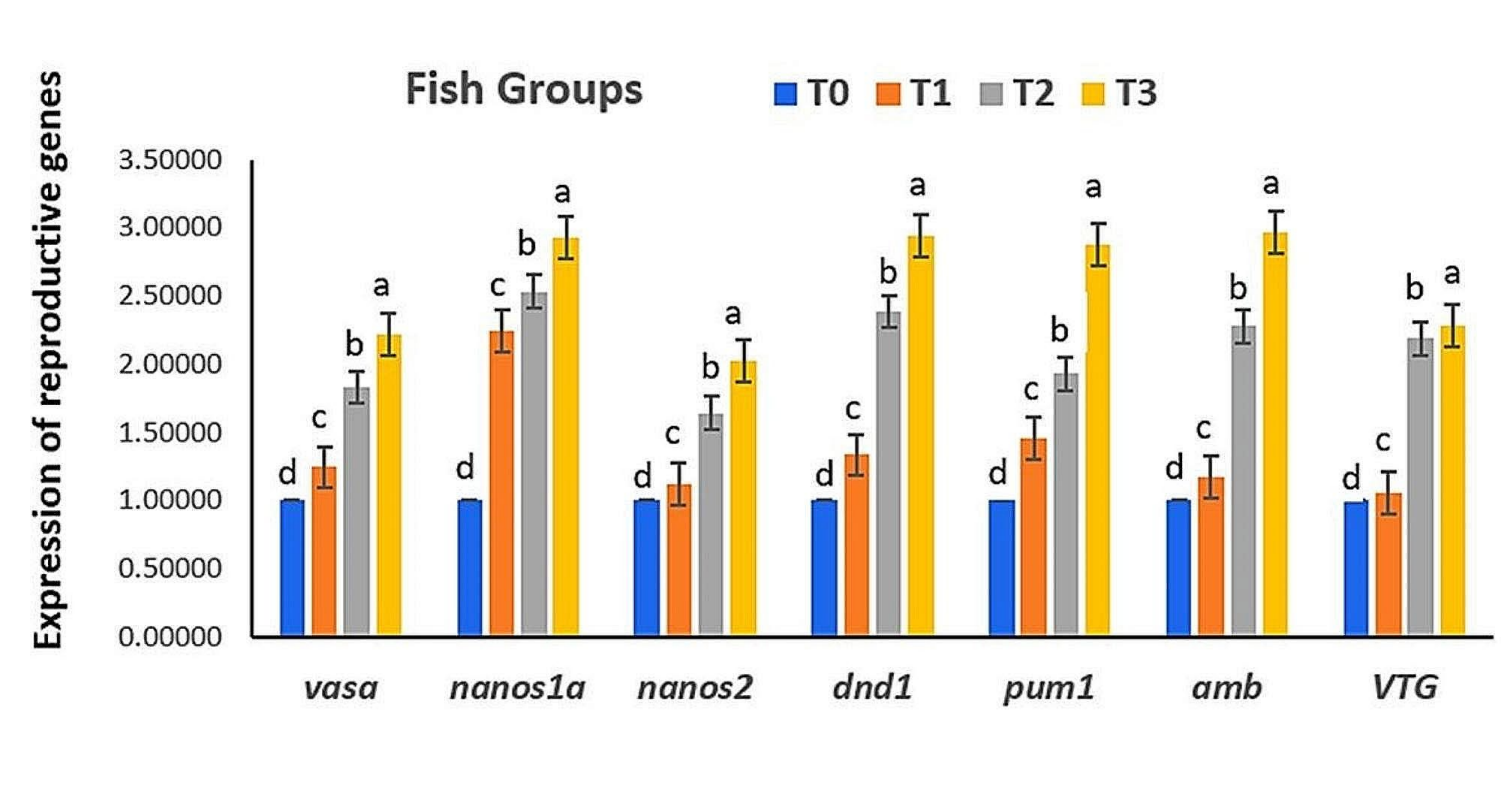
Effect of the *B. subtilis* and *B. licheniformis* (T0; 0, T1; 0.01, T2; 0.02, T3; 0.03, g/m^3^) added to the water on expression of reproduction-associated genes in the testis of Red Tilapia

**Fig. 2:**
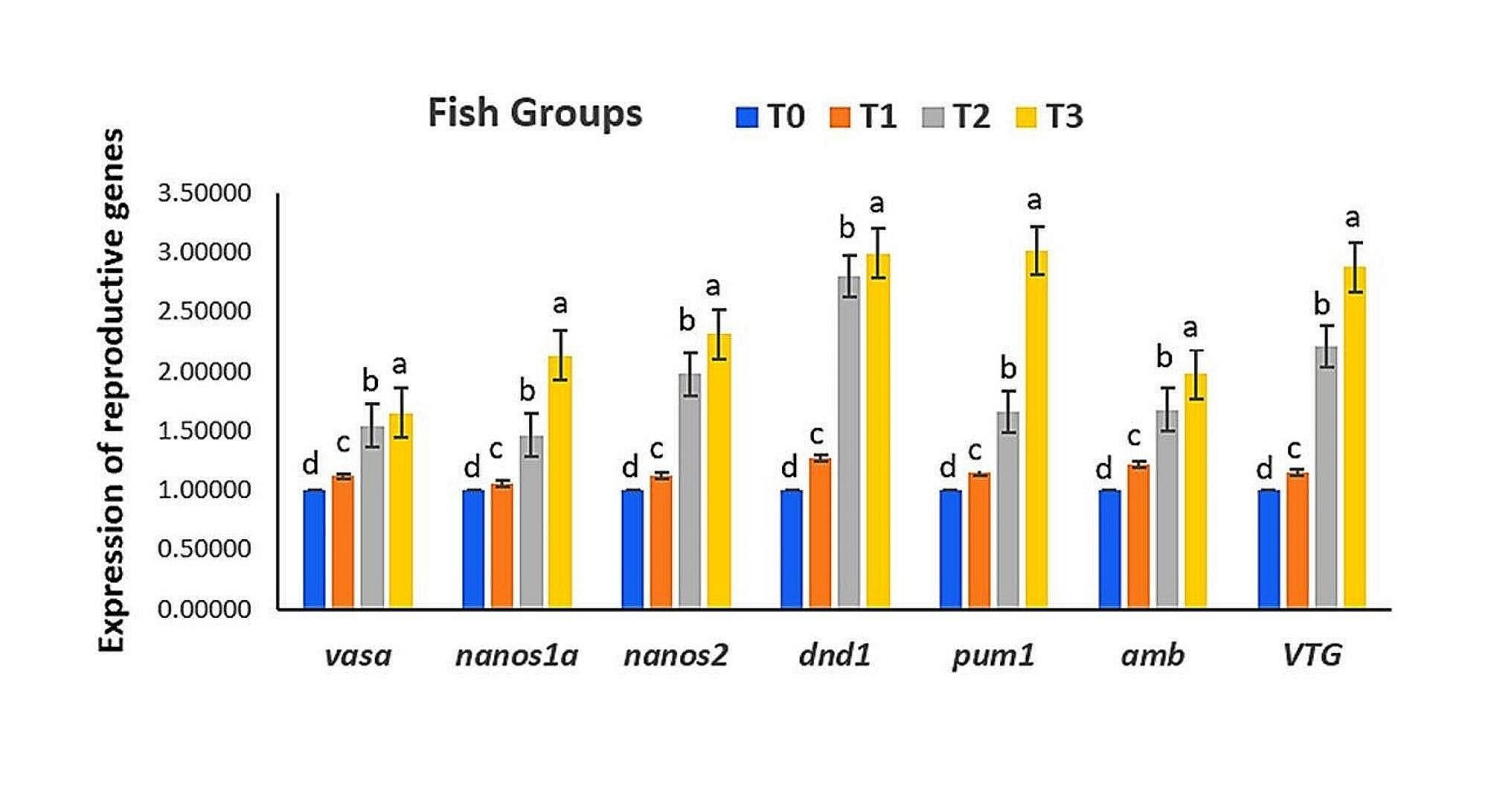
Effect of the *B. subtilis* and *B. licheniformis* (T0; 0, T1; 0.01, T2; 0.02, T3; 0.03, g/m^3^) added to the water on mRNA of reproduction-related genes in the ovaries of Red Tilapia

The current findings show the expression of genes associated with development and reproduction, including *Vasa*,* nanos1a*,* nanos2*,* dnd1*,* pum1*,* AMH*, and *VTG in* testicular (Fig. 1) and ovarian (Fig. 2) tissues of Red Tilapia. It was noted that the expressions of *Vasa*,* nanos1a*,* nanos2*,* dnd1*,* pum1*,* AMH*, and *VTG* genes in testicular tissues were significantly upregulated in response to different graded levels of *B. subtilis* and *B. licheniformis* (*P* < 0.05) compared to the control group (Fig. 1). Additionally, the expressions of *Vasa*,* nanos1a*,* nanos2*,* dnd1*,* pum1*,* AMH*, and *VTG* genes in the ovarian tissues followed the same pattern (Fig. 2). This upregulation increased in a dose-dependent manner, with levels of 0.03 g/m^3^ of *B. subtilis and B. licheniformis* being the most effective (Figs. 1 and 2).

### Histological changes in testicular tissue

**Fig. 3:**
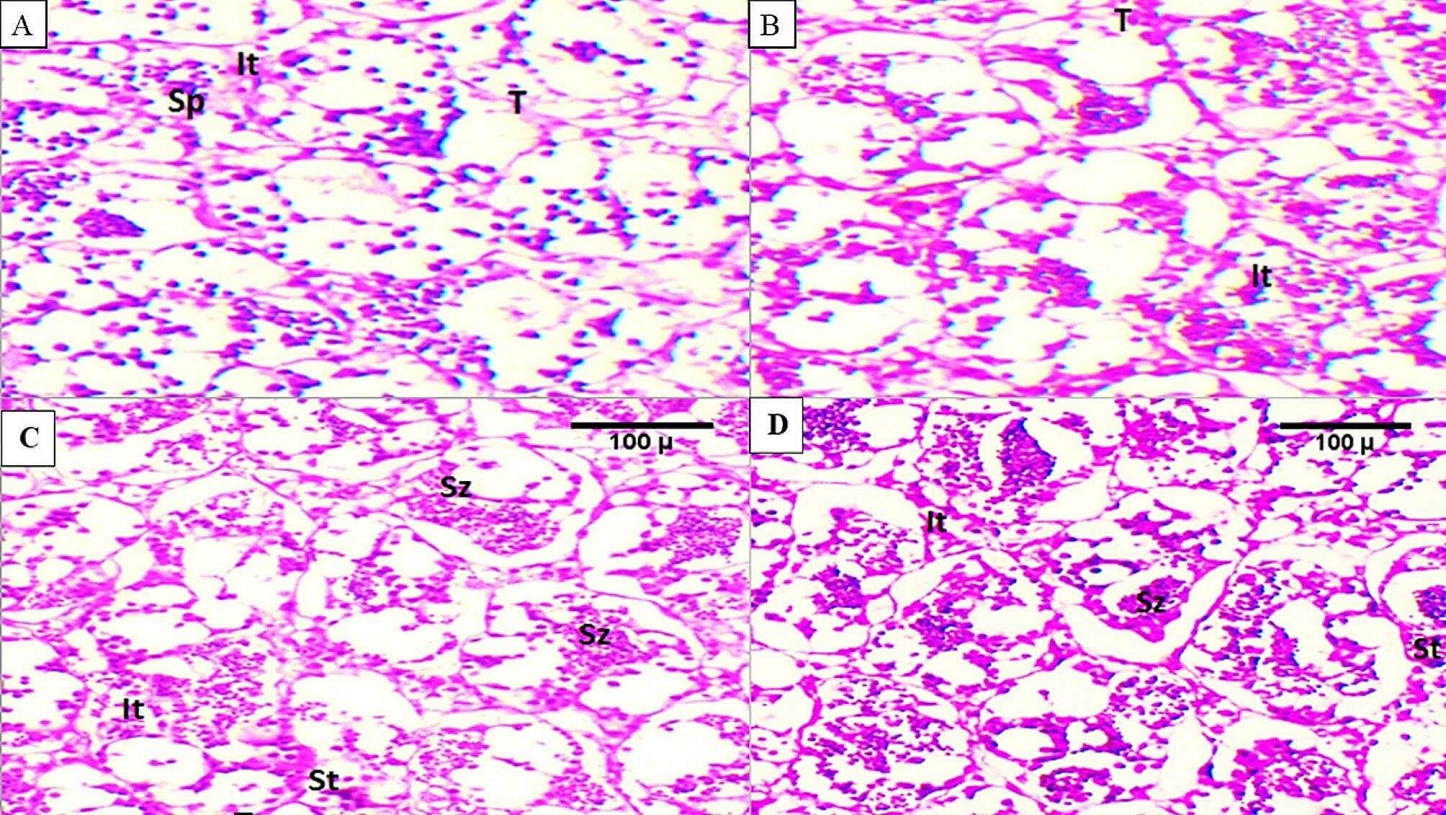
Photomicrographs of transverse sections of mature testis of Red Tilapia kept in various levels of *B. subtilis and B. licheniformis* {0 (Fig. 3**A**), 0.01(Fig. 3**B**), 0.02 (Fig. 3**C**), 0.03 (Fig. 3**D**, g/m^3^} added to the water. Interstitial cells (It), spermatocytes (Sp), spermatids (St), spermatozoa (Sz), testicular lobules (T). [H&E stain was used, 100 μm]

Sections of fish testicular tissue from the control group (T0; Fig. 3A) showed the typical anatomy of interstitial cells (It), spermatocytes (Sp), spermatids (St), spermatozoa (Sz), and testicular lobules (T). The testes treated with *B. subtilis* and *B. licheniformis* contained all stages of spermatogenesis. In the T1 group (Fig. 3B), which received 0.01 g/m^3^ of *B. subtilis* and *B. licheniformis*, we noticed normal and healthy architectures of seminiferous tubules containing spermatocytes, spermatids, and spermatozoa. There was a noticeable increase in the abundance of spermatogenetic cells and growth of testicular tubules, particularly in both the T2 (0.02 g/m^3^; Fig. 3C) and T3 groups (0.03 g/m^3^; Fig. 3D) treated with *B. subtilis* and *B. licheniformis*. The T3 group showed an increase in spermatogenic cells, particularly spermatids and mature spermatozoa (Fig. 3D).

### Histological changes in ovaries

**Fig. 4:**
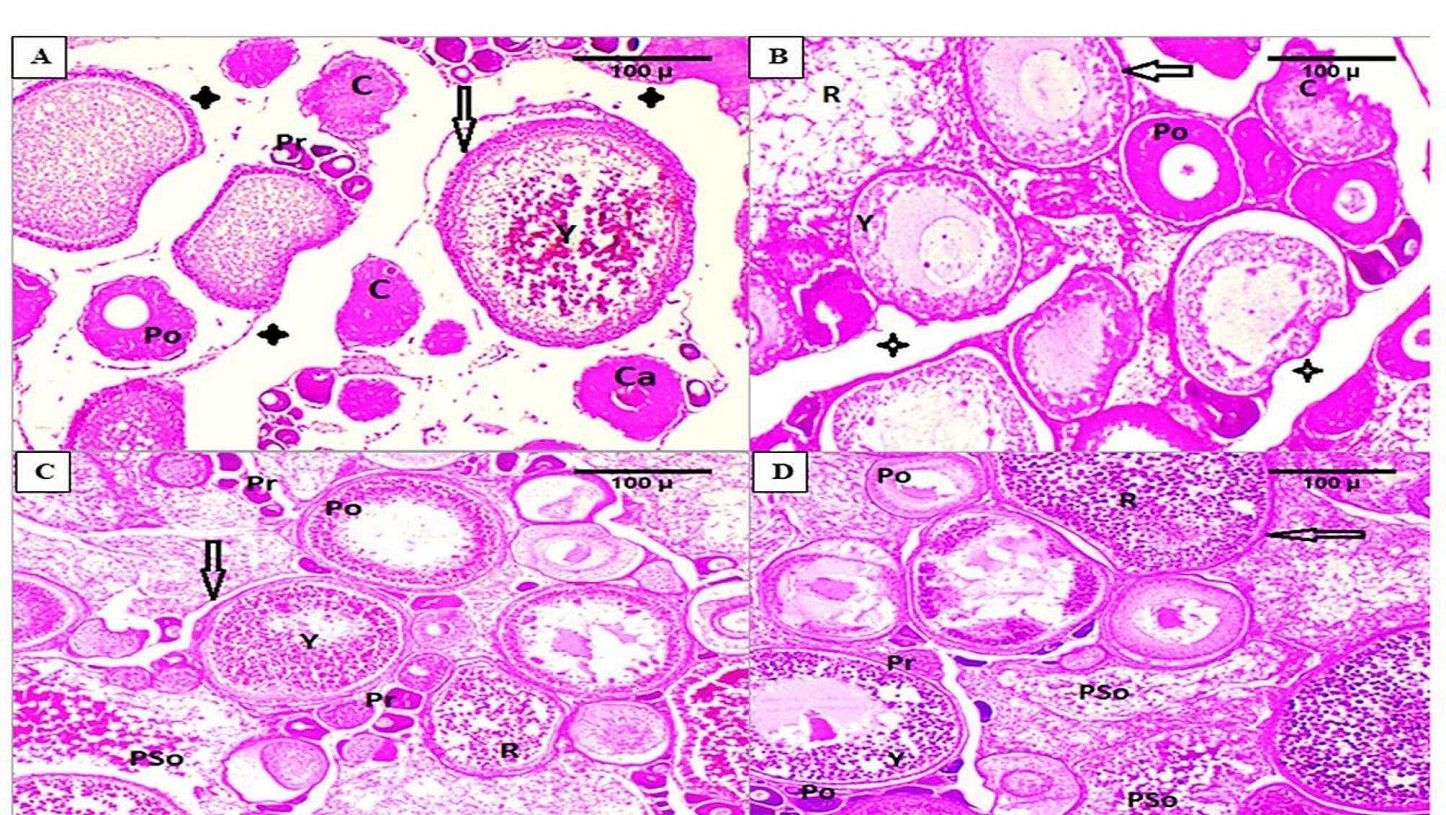
Photomicrographs of transverse sections of mature ovaries of Red Tilapia kept in water supplemented with various levels of *B. subtilis and B. licheniformis* {0 (Fig. 4**A**), 0.01 (Fig. 4**B**), 0.02 (Fig. 4**C**), 0.03 (Fig. 4**D**), g/m^3^}. Arrows: stroma that around the vitellogenic oocytes’ follicles, growing oocytes at different developmental stages, normal chromatin nucleolar oocyte (C), vitellogenic oocytes with cortical alveoli (CA), ripe oocytes (R), yolk globules (Y), previtellogenic stage (Pr), postvitellogenic stage (Po), postspawning ova are collapsed (PSo), asterisk: degeneration of some tissues around the oogonia follicles. [H&E stain was used, 100 μm]

The control group (T0) fish ovaries (Fig. 4A) displayed a slightly normal ovarian structure containing normal chromatin nucleolar oocytes (C), vitellogenic oocytes, cortical alveoli (CA), ripe oocytes (R), yolk globules (Y), and previtellogenic stage (Pr). Fish fed on different levels of *B. subtilis* and *B. licheniformis* (0.01, 0.02 and 0.03 mg/m^3^) exhibited normal development in all types of oocytes, including chromatin nucleolar oocytes (C), previtellogenic stage (Pr), vitellogenic oocytes, cortical alveoli (CA), ripe oocytes (R), yolk globules (Y), postvitellogenic stage (Po), and postspawning ova (PSo) (Fig. 4B). This development was most pronounced in the T2 (0.02 g/m^3^) and T3 (0.03 g/m^3^) groups. Compared to the control group (T0), the T2 (Fig. 4C) and T3 (Fig. 4D) groups showed an improvement in oocytes with post-ovulation luteinization and demonstrated superiority in oogonia and oocyte occurrence at various developmental stages.

## Discussion

Aquaculture has recently played a significant role as a vital food source, supplying humans with excellent protein and easily absorbable minerals, particularly in developing nations such as Egypt [20, 40, 41]. Enhancing fish broodstock reproductive capacity with probiotic supplements can benefit the industry’s sustainability. While most studies focus on probiotics’ role in growth stages, their impact on reproductive capacity is less explored. In this study, we investigated the effects of adding BSL to water on red Tilapia’s reproductive variables. The results show that supplementing water with BSL significantly improved hematobiochemical parameters, reproductive hormones, organosomatic attributes, and reproductive capacity in red Tilapia. Gene expression analysis revealed upregulation of reproductive-related genes in testicular and ovarian tissues in response to varying levels of B. subtilis and B. licheniformis compared to fish on a basal diet. Various reports have documented the positive impacts of different additives on fish. Among these additives, probiotics, especially Bacillus strains, have become the most widely used and popular in aquaculture [23, 42]. The production of fish in aquaculture heavily depends on water conditions. To achieve optimal reproductive capacity, survivability, and production, it is crucial to enhance the aquatic environment by reducing aquatic pathogens and improving water quality. This will lead to successful reproduction [6]. The findings of the current trial demonstrate a noticeable improvement in water quality, supported by a considerable decrease in total hazardous and toxic degrees of ammonia in the probiotics-treated groups, especially the T3 group. Many previous studies have found similar results [9, 43].

According to the findings of a research conducted by [44], the addition of *B. licheniformis* as denitrifying bacteria to rearing water decreases the levels of toxic components (TAN and NH_3_) and improves the breakdown of protein and starch in leftover feeds. The quality of water is enhanced by the biodegradation of nitrogenous wastes by Bacillus species, resulting in waste mineralization [44]. Maintaining good water quality is crucial for the survival of aquatic organisms especially in Broadstock fish, with ammonia nitrogen and nitrite nitrogen being key indicators in aquaculture. High levels of these compounds can be toxic to farmed species. Effective water quality management is essential in aquaculture production. Enriching water with efficient microbial communities can enhance organic matter recycling and maintain a clean water environment for farmed fish [45]. Previous studies have found that the addition of *B. subtilis* (10^9^ CFU/mL) significantly decreased the total nitrogen and ammonia nitrogen concentrations in water. Additionally, Cha et al. [46], performed that *B. subtilis* (0.5% of the diet) effectively reduced the concentration of ammonia nitrogen in the Japanese flounder (*Paralichthys olivaceus*) culture system. The authors suggested that probiotics play a crucial role in water quality by breaking down organic matter and converting NH^4+^ to NO^3^. Furthermore, probiotics have been shown to eliminate pathogenic bacteria from water. Improving water quality can enhance fish health, leading to increased production and reduced susceptibility to disease.

Haemato-biochemical parameters are considered valuable indicators for evaluating the health profile of fish [47, 48]. According to our findings, the use of *B. subtilis* and *B. licheniformis* improved the hematopoietic state of red tilapia. Hematological parameters in the current study, such as HB, mean MHC, MCHC, and HCT in the treated groups with the addition of probiotics also significantly increased compared to the control group, indicating a high capacity for oxygen carrying in the blood [49]. On the contrary, the MCV was not significantly affected in all treatments. The addition of *B*. *subtilis* in water demonstrated a significant improvement in albumin, total protein, and globulin values compared to the control group [50, 51]. Glucose levels in our study exhibited a gradual decline in all treated groups, which is in line with the results of a previous study [52]. The reduction in glucose levels was attributed to the probiotic’s treatment altering the expression of genes involved in glucose uptake and lowering overall glucose levels in zebrafish larvae [52]. Significant differences were observed in the blood serum composition of red tilapia that received supplementation with *B. licheniformis* and *B. subtilis*. Components in the blood serum indicate the physiological performance of the fish body, especially in relation to the functions of vital organs such as the liver, kidneys, and the circulatory system. Hepatic function enzymes, AST, and ALT are biochemical indices of liver function and health. These indicators are used to evaluate how additives can influence the metabolic activities and overall health of fish [41]. In our trial, a significant reduction in liver activities was observed in all groups treated with *B. subtilis* and *B. licheniformis*, which is consistent with findings reported in Nile tilapia showing the same effect of these enzymes when probiotics are added to the rearing water.

The part of probiotics in controlling metabolic enzymes has also been explored and studied in a scarce other aquatic fish species. Studies by [53] and [54] suggested that feeding Nile tilapia a diet supplemented with *B. subtilis* may reduce ALT and AST activities.

In the current research, we noticed a significant decrease in blood levels of creatinine, urea, and uric acid levels showed a significant decrease with the increase in probiotic levels. This data was in contrast to the findings by [55] and [20], who indicated that no notable changes in creatinine and urea levels among supplemented groups with probiotics. Additionally, the activities of antioxidants SOD and CAT were increased with the addition of graded levels of *B. subtilis* and *B. licheniformis* (*p* < 0.05), consistent with the results of study by [20]. Prebiotics and probiotics have been stated to improve the reproductive capability of certain fish species. For instance, Zebrafish (*Danio rerio*) fed a diet enriched either *bacillus* spp or *lactobacillus* spp showed improvements in gonad development, fecundity, egg production, GSI, and the number of viable fries produced [56, 57].

As a secondary effect of increased absorption and utilization of nutrients in aquatic animals receiving probiotic supplementation, there is an increased availability of nutrients essential for reproductive system function, including the production of hormones important for reproductive function. Pituitary gonadotropins (GnRH) such as LH and FSH are the main regulators of gametogenesis in teleost fish [58].

The data of the current experiment is consistent with the findings by [27], indicating that red tilapia receiving probiotic supplementation will experience an increase in the production of hormones such as testosterone, FSH, LH, estrogen, and progesterone compared to the control group.When using probiotics for aquatic animals, the type of probiotic bacteria and the dosage of probiotics play a significant role in the outcomes. In this study, the T3 treatment group (dose 0.03 g/m^3^) exhibited a greater increase in hormone concentrations compared to the T2 and T1 treatment groups [27]. The results of this experiment showed that the treatment with three levels of probiotics containing *B. subtilis* and *B. licheniformis* had a significantly greater effect on the reproductive hormones of red tilapia compared to the fish fed the basal diet without any treatment.

Specifically, the probiotic treatment at level 3 (T3) produced highly significant results in increasing the concentration of the hormones FSH, LH, E2, and P compared to the other treatment groups. Regarding testosterone hormone, the highest level was found in the T3 treatment, while for T1 and T2, they did not differ significantly different from the control group (T0).

Studies by several researchers [48, 59, 60] have documented that beneficial microbes can lead organisms utilize energy sources more efficiently, leading to improved growth and reproductive performance in zebrafish [61]. In our study, the inclusion of levels of *B. licheniformis* and *B. subtilis* notably enhanced the growth performance of red tilapia compared to the control group.

Furthermore, higher levels of these probiotics in red tilapia groups reared in water treated with *B. subtilis* and *B. licheniformis* can be directly contributed to the improvement of water quality. There was a substantial variation (*P* < 0.05) between the treatment groups in the GSI percentage, mean number of fries, and mean fry weight parameters. Only the group receiving probiotic supplementation showed a significant difference in the HIS percentage and egg diameter parameters compared to the control group. Body indices, including GSI, VSI, and HSI, which indicating dietary value, growth, and feed utilization, can be improved by adding feed with a mixture of *B. subtilis* and *B. licheniformis* [62]. Another study of [62] reported that all doses of probiotics had a substantial valuable consequence on the GSI and HSI indices compared to the control treatment. This suggests that adding *B. licheniformis* and *B. subtilis* can boost the reproductive capability of zebrafish [56, 57].

*Dead-end (dnd)*,* Nanos*,* pumilio (pum)*,* piwil-like (piwil) vasa*, and genes are known to be implicated in translational repression of germ cells [63], which is believed to be essential for the preservation of germline integrity across animal phyla, containing mice [64], zebrafish [65], and Xenopus [66]. Recently, four *nanos’* genes [67], two *piwil* genes [68] and three *vasa* genes [69] have been identified in tilapias. Additionally, *in silico* examination of public databases by NCBI revealed anticipated sequences for three pum genes and one *dnd1a*. Vitellogenin (Vtg) is a reproductive protein found in females, that is broken down into yolk proteins. Lipovitellin (Lv), and phosvitin (Pv), which are deposited in eggs to provide essential nutrients for early-stage embryos. Several studies have confirmed that probiotics can improve the reproductive capacity in Nile tilapia by supporting reproductive-related genes, as observed in this study. A study by [70] clarified that probiotics (0.5 g/kg) added to Nile tilapia feed during the breeding season improved reproductive performance and profitability.

In the extant work, the transcript of development-reproduction-related genes in red tilapia fed with *B. subtilis* and *B. licemiformis* were significantly upregulated compared to fish reared in the control group. This highlights the beneficial effects of *B. subtilis* and *B. licemiformis* on fish reproduction, in addition to the previously reported improvements in hematology profile, blood metabolites, and reproductive parameters such as GSI, egg diameter, and fry production. Male zebrafish fed a diet with a containing probiotic *P. acidilactici* exhibited higher expression of fertility markers (*lepa*,* dmrt*,* and bdnf*) compared to the control group [71]. This indicates that *P. acidilactici* could be a promising probiotic supplement to enhance molecular parameters in testicular cells of male zebrafish, potentially leading to improve the reproductive performance, sperm quality.Probiotics have been shown to prevent apoptosis and enhance survivability in fish during the growth period [72]. They also stimulate the intratubular and tubular sections, which are known to enhance sperm production [71]. Certain probiotics have been shown to activate various cell types, including neuronal, connective tissue, blood/lymphatic vessels, mast cells, macrophages, and steroidogenic Leydig cells. Additionally, probiotic supplementation in feed has been found to improve fish reproductive health and feed utilization, particularly with lactic acid bacteria [24].

Probiotics have the potential to modulate gene expression patterns or hormone levels that regulate fish reproduction [73], thereby enhancing reproductive functions and activating reproductive genes to address reproductive disorders when added to the diet or water. Histological investigations revealed that the addition of *B. subtilis* and *B. licemiformis* enhanced gonadal development in red tilapia, particularly in spermatogenic cells, including spermatids and mature spermatozoa. Female fish reared in 0.02 and 0.03 g/m^3^ showed different stages of oocyte development, with the best gonadal development observed in the 0.03 g/m^3^ group, which had a higher number of mature and ripe oocytes. These findings are consistent with those reported by [74] in Nile tilapia. Further studies are needed to confirm these results, as there is a lack of research on the potential effects of probiotics on reproductive performance in fish species, especially using omics tools.

## Conclusion

The study showed that adding *B. subtilis* and *B. licheniformis* at a concentration of 0.03 g/m^3^ can enhance fish blood profile and reproductive health. This experiment demonstrated that probiotics in water can improve water quality, hematological and biochemical parameters in red tilapia broodfish, and support gonad maturation, gametogenesis production, gene expression, and overall reproductive performance. Additional research is required to validate these findings, as there is a dearth of studies examining the potential impacts of probiotics on reproductive performance in fish species.

## Data Availability

No datasets were generated or analysed during the current study.
